# Webbed skin of the posterior labial commissure: A rare cause of dyspareunia: A case report

**DOI:** 10.1097/MD.0000000000042731

**Published:** 2025-06-20

**Authors:** Shoujie Wang, Kai Xu, Banghui Shi, Zhikang Zhu, Jialun Shen, Xiaoling Chen

**Affiliations:** a Department of Plastic Surgery, The Fourth Affiliated Hospital of School of Medicine, and International School of Medicine, International Institutes of Medicine, Zhejiang University, Yiwu, China; b Department of Plastic Surgery, The First Affiliated Hospital, School of Medicine, Zhejiang University, Hangzhou, China.

**Keywords:** dyspareunia, five-flap plasty, webbed skin of posterior labial commissure

## Abstract

**Rationale:**

Dyspareunia is a common disease affecting the physical and mental health of individuals. There are several reasons for dyspareunia. It is essential to identify the causes of dyspareunia and to implement targeted treatments.

**Patient concerns:**

The patient was a 26-year-old married woman who presented with dyspareunia lasting for several months, and never successfully engaged in sexual intercourse. She had a webbed skin with a scar at the posterior labial commissure from the 5 o’clock position to the 7 o’clock position of the vaginal opening, partially blocking it.

**Diagnoses:**

This case presented a rare cause of dyspareunia.

**Interventions:**

In this case, we designed a five-flap method to extend the skin length of the vaginal opening and solved the problem of obscuration of the patient’s vaginal opening.

**Outcomes:**

After surgery, the vaginal opening appeared normal, with an apparent increase in diameter, and the incision was recovered.

**Lessons:**

In this case, we attempted a five-flap plasty on the webbed skin of the posterior labial commissure, and the outcome was satisfactory. This demonstrates the potential of this new treatment. This case report can help clinicians broaden their treatment options.

## 1. Introduction

Dyspareunia is described as a pain associated with sexual intercourse. It occurs frequently. In 2006, the prevalence of dyspareunia ranged from 8% to 21.1%, with global variation.^[1]^

There are many causes of dyspareunia; however, the leading ones include postpartum dyspareunia, endometriosis, insufficient vaginal lubrication or arousal, anogenital causes, and psychological causes.^[2]^

The management of dyspareunia depends on the cause of pain.^[3]^ Therefore, identifying the cause of dyspareunia and implementing targeted treatment are crucial.

Herein, we present the case of a woman with dyspareunia associated with pain upon attempting vaginal penetration.

## 2. Case report

We present the case of a 26-year-old married woman who presented with dyspareunia that lasted for several months. The patient never successfully engaged in sexual intercourse. She and her husband tried it several times, but all failed. The patient was born with an uncommon webbed skin of the posterior labial commissure. She experienced pain in the vaginal opening and sometimes bleeding, ledding to the formation of scars here and intensified the pain. The patient had no other symptoms including abnormal vaginal discharge, mental disorders, dysuria, dysmenorrhea, or pelvic pain.

Diagnosis can be made through physical examination, ultrasound, and other imaging techniques such as CT and MRI to determine whether there are any lesions in the skin, or any associated tumors, or any structural abnormalities.

**Figure 1.**
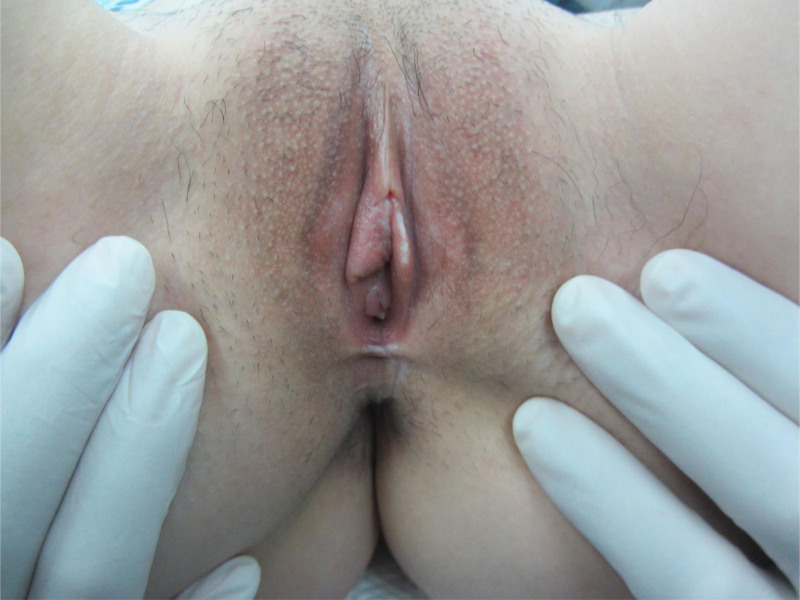
Before operation. The webbed skin with a scar of the posterior labial commissure was observed from the 5 o’clock position to the 7 o’clock position of the vaginal opening, partly blocking the vaginal opening.

On examination, webbed skin with a scar on the posterior labial commissure was observed from the 5 o’clock position to the 7 o’clock position of the vaginal opening, partially blocking it. The results of other vaginal examinations were normal (Fig. 1). The patient experienced intense pain while her husband attempted vaginal penetration.

**Figure 2.**
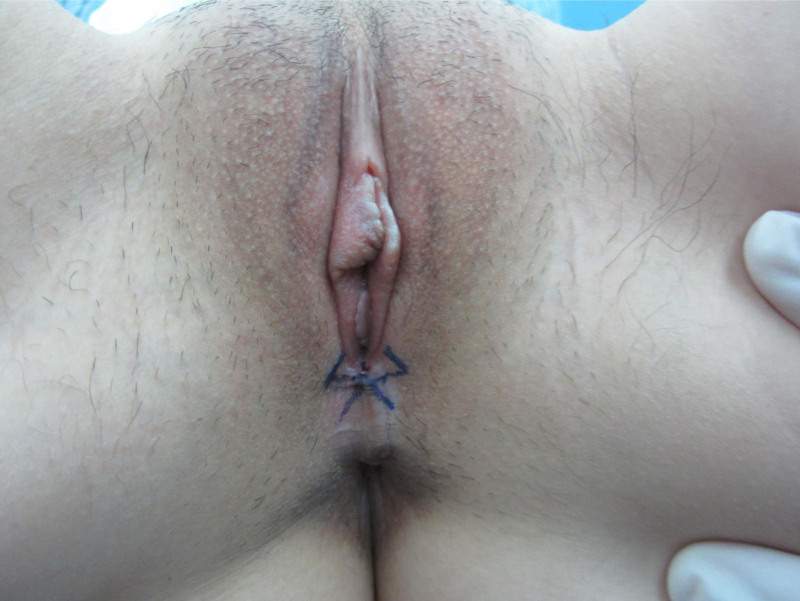
Design of the “five-flap” plasty.

**Figure 3.**
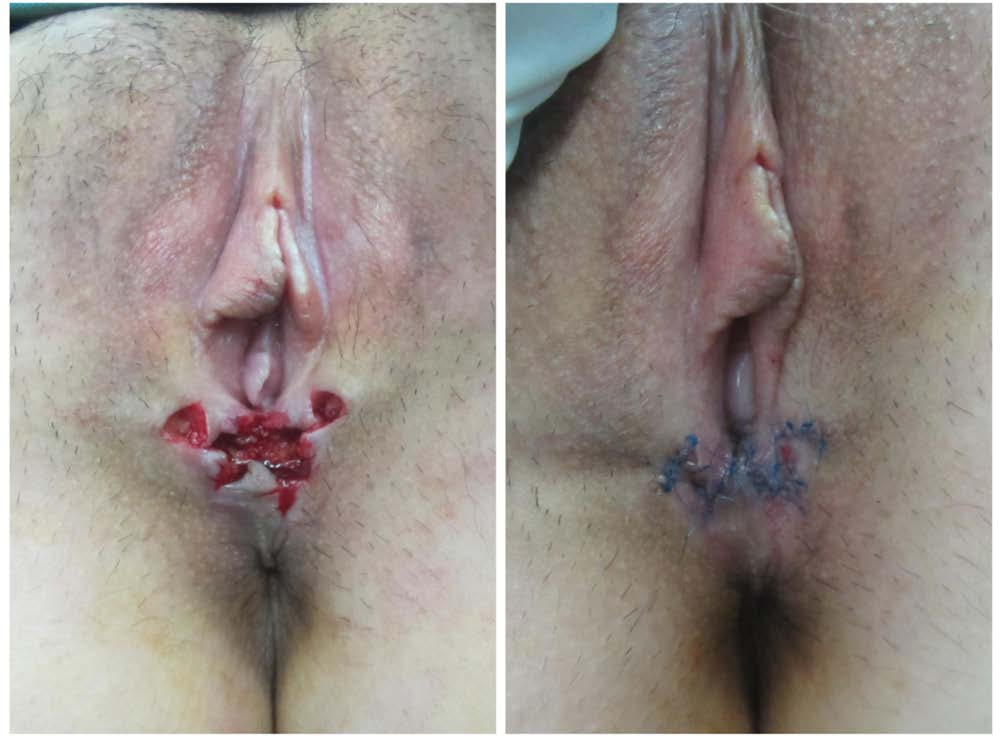
During and after surgery.

The patient underwent surgical treatment based on the vaginal examination findings. We designed the “five-flap” plasty to relieve the pull (Fig. 2), widen the vaginal opening, and improve the patient’s symptoms. During surgery, each flap was fully separated, and after surgery, the length of the vaginal opening increased (Fig. 3).

**Figure 4.**
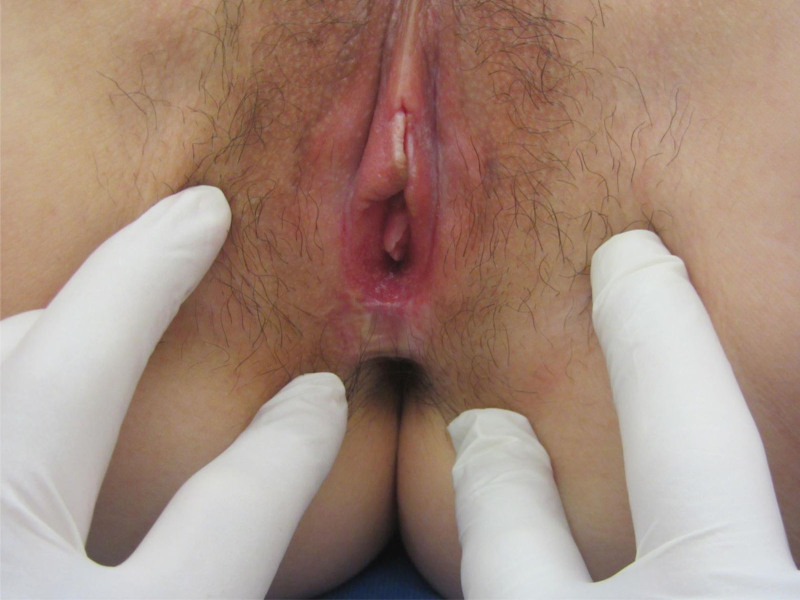
Six months post-surgery. The incision had healed well.

Twelve days after the operation, the wound of the patient was completely healed and stitches were removed. Three months after the operation, the patient showed no obvious scar, no scar contracture, no obvious skin pulling of the vaginal opening. Six months after surgery, the vaginal opening appeared normal with an apparent increase in diameter, and the incision was recovered (Fig. 4). Dyspareunia was resolved, and the patient successfully achieved sexual intercourse. About 1 year after surgery, the patient was pregnant naturally.

Some possible complications of flap plasty include flap necrosis, hematoma formation under the flaps, wound infection, and scar contracture recurrence.^[4]^ None of the above complications occurred.

During recovery, the patient felt anxious and tense about potential complications and surgical failure. Thankfully, the successful operation led to a smooth recovery, which yielded satisfactory results.

## 3. Discussion

Dyspareunia is a common but poorly understood condition affecting many sexually active women. It refers to recurring or persistent pain associated with an attempt or complete vaginal penetration or penile-vaginal intercourse.^[3,5]^

The causes of dyspareunia vary. Here, we present a rare etiology of dyspareunia. The patient was born with an uncommon webbed skin of the posterior labial commissure, causing pain during sexual intercourse due to obstruction of the vaginal opening. There had no history of this symptom. There was no history of trauma, disease, or cancer in the pelvic region. This is a congenital condition. No specific illness or psychological factor was associated with the patient’s condition.

Injury to the perineum is an important cause of dyspareunia, especially in postpartum patients.^[6,7]^ Postpartum scar in the perineal area can lead to pain during intercourse, resulting in dyspareunia.^[7,8]^ Scar tissues resulting from lacerations or episiotomies during labor can affect sexual intercourse, which commonly requires specific surgical intervention.

Herein, the patient presented with a congenital obstruction in the vaginal opening and attempted multiple unsuccessful sexual intercourses. This causes skin damage resulting in pain, bleeding, and scarring.

The webbed skin with a scar on the posterior labial commissure blocked the vaginal opening. The deformity obstructed the penile-vaginal intercourse and reduced the patient’s quality of life.

The conventional surgical procedure involves incising the skin and then suturing; however, we used a different approach to repair the deformity: five-flap plasty.

Five-flap plasty is a common flap repair procedure that can improve cicatricial contracture deformities of various body parts. The procedure uses 2 Z-plasties and one Y-V plasty, and is used at different anatomic sites to release scar contracture.^[9]^

In 1959, Mustarde introduced a five-flap plasty to repair epicanthal folds caused by blepharophimosis.^[10]^ An opposing study documented double opposing Z-plasty, a modification of five-flap z-plasty, to correct epicanthic folds,^[11]^ which was subsequently modified by Hirshowitz and named the five-flap procedure for correction of the axillary web and web contractures of the hand.^[12–14]^ Many modified five-flap plasties have been used to restore contractures.^[15–17]^

Currently, this method is used to treat scars or skin contractures. The classic five-flap plasty has a theoretical advantage of approximately 150%, in terms of skin contracture and scar length.

**Figure 5.**
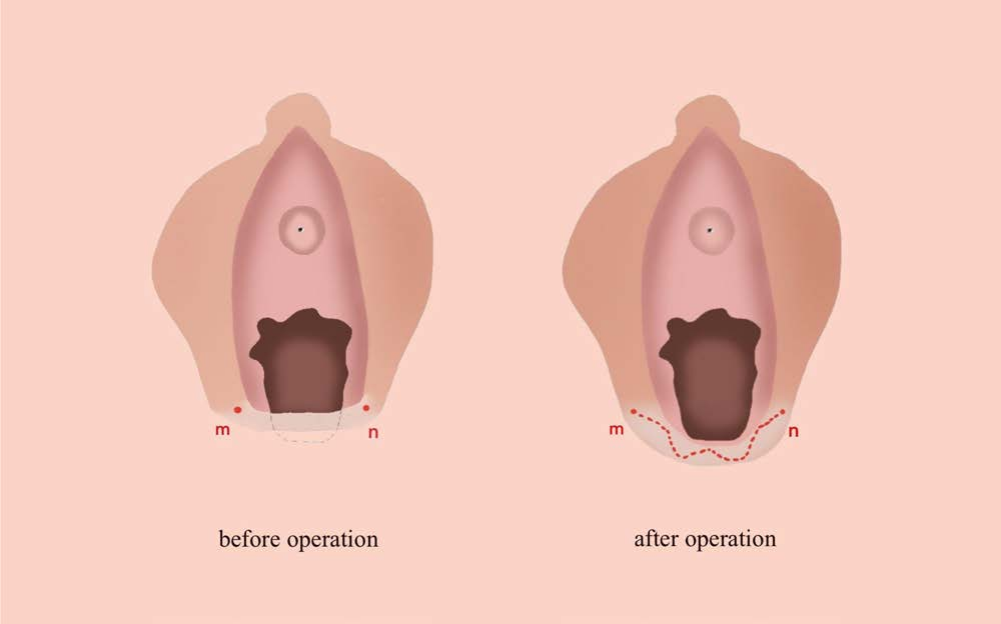
The vaginal opening was partially blocked by webbed skin with scar (left). The length of vaginal opening was increased apparently with a curved incision (right). The axis of five-flap Z-plasty run along the contracture lines. (left “mn”). The incision was curved after operation (right “mn”).

**Figure 6.**
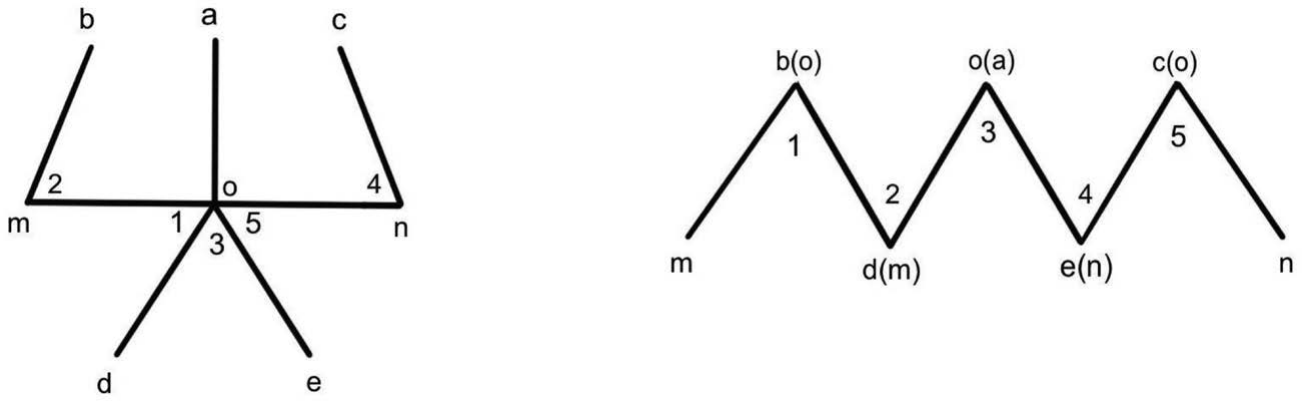
Supposing ∠ 1 = ∠2 = ∠3 = ∠4 = ∠5 = 60° and de = oe = od = om = on = mb = nc(left). After incision, 5 triangular flaps (∠1–5) were formed, and the flaps were recombined(right). Original length of “mn” was “2de” (left “mn”), the total length after operation of “mn” was about “‘3de’” (right “mn”).

In this case, the vaginal opening was blocked by webbed skin of the posterior labial commissure. To treat this, we designed a classic five-flap method using the contracture line as the axis (Fig. 5, left “mn”) and designed 2 “Z” plasties and a “Y-V” plasty, with a 60-degree angle and approximately equal length of each incision line to extend the skin length of the vaginal opening by about 1.5 times (Fig. 5, right “mn”). We now provide a step-by-step description of the incision design, including diagrams to illustrate the specific techniques used (Figs. 5 and 6). The five-flap plasty results in a curved incision. Curved or zigzag incisions can better relieve contracture scars.^[18–20]^ The conventional surgical methods consists of a longitudinal incision and then a crosswise suture. Compared with the method, the five-flap plasty technique can significantly enhance the length of the vaginal opening, reduce skin tension, and decrease the possibility of postoperative contracture. Other methods include Z plasty, V-Y plasty, or other flap plasty. The five-flap plasty is essentially a modified flap plasty, it consists of 2 “Z” plasties and a “Y-V” plasty. Compared with other flap, the five-flap plasty technique can obtain more length gain.^[15,21]^

## 4. Conclusion

Our case demonstrates a rare cause of dyspareunia and provides an effective treatment option.

The patient had congenital webbed skin pulling of the vaginal opening which shielded the vaginal opening, thereby affecting sexual intercourse. We extended the vaginal opening by using the five-valve method.

Flaps are commonly used for wound healing, whereas five-flap plasty is used for contracture scars and skin repair in various body parts; however, perineal application is rare. We attempted to use it for webbed skin of the posterior labial commissure, and the outcome was satisfactory.

## Author contributions

**Conceptualization:** Shoujie Wang, Xiaoling Chen.

**Data curation:** Banghui Shi.

**Formal analysis:** Banghui Shi.

**Investigation:** Kai Xu.

**Project administration:** Xiaoling Chen.

**Supervision:** Jialun Shen.

**Validation:** Jialun Shen.

**Writing – original draft:** Shoujie Wang, Kai Xu.

**Writing – review & editing:** Zhikang Zhu, Xiaoling Chen.
